# Gallbladder Gallstone Disease Is Associated with Newly Diagnosed Coronary Artery Atherosclerotic Disease: A Cross-Sectional Study

**DOI:** 10.1371/journal.pone.0075400

**Published:** 2013-09-18

**Authors:** Zhao-Yan Jiang, Xia Sheng, Chen-Ying Xu, Wei-Wei Li, Xian-Xing Chang, Lu-Ying Sun, Xiao-Bo Yang, Li-Fen Yu

**Affiliations:** 1 Department of Surgery, Shanghai Institute of Digestive Surgery, Ruijin Hospital, Shanghai Jiao Tong University School of Medicine, Shanghai, China; 2 Department of Gastroenterology, Ruijin Hospital, Shanghai Jiao Tong University School of Medicine, Shanghai, China; 3 Division of Biostatistics, Center of Service Science, Shanghai Advanced Research Institute, Chinese Academy of Science, Shanghai, China; King’s College London School of Medicine, United Kingdom

## Abstract

**Background and Aims:**

The association between gallstone disease and coronary artery atherosclerotic disease (CAD) remains unclear. To clarify their relationship, patients with CAD newly diagnosed by coronary angiography were investigated in this cross-sectional study.

**Methods:**

The study cohort consisted of 1,270 patients undergoing coronary angiography for the first time between January 2007 and September 2011. Patients with ≥50% diameter stenosis in any major coronary artery on coronary angiography were defined as being CAD positive (n = 766) and those with no stenosis as CAD negative (n = 504). Multivariate logistic regression was used to investigate the relationship between gallstone disease and CAD. The odds ratios (OR) of factors associated with CAD were calculated. In addition, CAD-positive and CAD-negative patients were matched one-to-one by age, gender and metabolic syndrome (MetS), and the association between gallbladder disease and CAD was determined.

**Results:**

The prevalence of gallstone disease was significantly higher in CAD-positive than in CAD negative patients (149/766 [19.5%] vs 57/504 [11.3%], P<0.01). Gallstone disease was significantly associated with CAD (adjusted OR = 1.59, 95% confidence interval [CI] 1.10–2.31). Following matched pairing of 320 patients per group, gallstone disease remained significantly associated with CAD (adjusted OR = 1.69, 95% CI: 1.08–2.65).

**Conclusion:**

Gallstone disease is strongly associated with CAD diagnosed by coronary angiography.

## Introduction

Coronary artery atherosclerotic disease (CAD), defined as the blockage of one or more coronary arteries, is common throughout the population, which is usually caused by atherosclerosis. It is one of the leading worldwide causes of death and disability [1]. Gallstone disease is also common world-wide [2]. In China, its prevalence has increased in recent years. Our survey of residents of Shanghai found that 10.8% of subjects had gallstone disease and 2.97% had a previous cholecystectomy (Jiang ZY, unpublished data), which is much higher than the recent reported prevalence [3].

CAD and gallstone disease both result from the accumulation of cholesterol, in the coronary artery walls and gallbladder cavity, respectively. These conditions share several common risk factors, including age, gender, overweight and disorders of lipid and glucose metabolism [4]. These factors are also key components for the diagnosis of metabolic syndrome (MetS) [5]. MetS is highly associated with CAD [4], and gallbladder stones can be considered a biliary feature of MetS. Furthermore, the association of gallstone disease and non-alcoholic fatty liver disease (NAFLD) has also drawn much attention by researchers in this field [6].

Since the first study of the relationship between gallstone disease and cardiovascular disease in 1954 [7], many studies have analyzed the association between these two conditions, with conflicting results [8]–[11]. For example, a critical review found no strong association between coronary heart disease and gallbladder disease [8], whereas a 26-year follow-up of the Framingham Heart Study found that cholecystectomy was associated with coronary heart disease, but only in men [9]. In another study, although gallbladder disease was found to be associated with ischemic heart disease, this association disappeared when only patients who had undergone cholecystectomy were included [10]. An analysis of Japanese patients found that the risk of death from coronary heart disease was higher in patients with cholesterol gallstone than pigment gallstone disease, but the difference was not statistically significant [11]. In contrast, the risk of death from stroke was significantly lower in patients with cholesterol than pigment stones.

One drawback of those studies was the definitions of cardiovascular disease and coronary heart disease, both of which comprise a series of diseases that overlap. These studies included patients with CAD, which is mainly caused by disorders of cholesterol metabolism, as well as patients with diseases other than CAD caused by congenital conditions and cardiomyopathy. The current gold standard in the diagnosis of CAD is coronary angiography. Due to the invasive nature of coronary angiography, however, no study to date has ever been able to analyze the association between gallstone disease and CAD.

In this cross-sectional study, we analyzed patients undergoing coronary angiography for the first time to explore the relationship between gallstone disease and newly diagnosed CAD. To minimize the influence of confounding factors, such as age, gender and MetS, we matched patients with and without CAD one-to-one and analyzed the association between gallstone disease and CAD in these matched pairs.

## Subjects and Methods

This cross-sectional study was performed in the cardiovascular clinic at the Ruijin Hospital in Shanghai, China, from January 2007 to September 2011. The study protocol was approved by the Ethics Committee at Ruijin Hospital. Written informed consent was obtained from each patient.

### Study Population/Patients

The study population consisted of patients undergoing coronary angiography for the first time at Ruijin Hospital for suspected coronary artery disease. To minimize the influence of lifestyle and dietary habits among different regions, only patients who were native to or lived in Shanghai city for more than 5 years were included. In accordance with American College of Cardiology/American Heart Association guidelines, patients with ≥50% diameter stenosis in any major coronary artery on coronary angiography were considered CAD positive; patients with no stenosis were considered CAD negative. Patients with stenosis >0% but <50% were excluded because their lesions may progress, and patients without significant obstructions may have more cardiovascular events during follow-up than patients with truly normal coronary angiograms [12]. Patients who underwent coronary angiography for other reasons, such as congenital heart disease, congestive heart failure, cardiomyopathy and vascular heart diseases, were excluded.

### Diagnosis of Gallstone Disease

Gallstone disease was confirmed by B-type ultrasonography, using the same criteria as reported [13]. Patients with previous cholecystectomies were not included because of uncertainty whether their indications for surgery were gallstone disease or other gallbladder diseases. Although we were unable to measure the cholesterol content of gallstones, our recent study of 104 gallstone samples from patients in Shanghai who underwent laparoscopic cholecystectomy found that 79.4% of the gallstones were of the cholesterol type (Jiang ZY, unpublished data). Thus, we assumed that most of the gallstones in our population were of the cholesterol type similar as that has been reported in western countries [14].

### Metabolic Syndrome Assessment

Patients were included in this study only if they were clearly found to have or not have MetS according to published criteria [5]. MetS was defined as having three or more of the following criteria [5]: (a) waist circumference ≥90 cm for males and ≥80 cm for females; (b) serum triglyceride (TG) concentration ≥150 mg/dL (≥1.7 mmol/L), or drug treatment for elevated triglycerides; (c) high density lipoprotein cholesterol (HDL-C) concentration <40 mg/dL (<1.0 mmol/L) for males and <50 mg/dL (<1.3 mmol/L) for females, or drug treatment for reduced HDL-C; (d) systolic blood pressure ≥130 mmHg and/or diastolic blood pressure ≥85 mmHg, or antihypertensive drug treatment in patients with a history of hypertension; and (e) fasting glucose concentration >100 mg/dL (>5.6 mmol/L) or drug treatment for elevated glucose. Waist circumference was measured and body mass index (BMI) was calculated from the height and weight of each individual. Fasting serum concentrations of total cholesterol (TC), low density lipoprotein cholesterol (LDL-C), HDL-C, TG, fasting glucose and HbA1c were also measured.

### Diagnosis of Non-alcoholic Fatty Liver Disease and Diabetes Mellitus (DM)

The diagnostic criteria for NAFLD on B-type ultrasound included diffusely increased echogenicity (“bright”) of the liver, liver echogenicity greater than kidney echogenicity, vascular blurring, and deep attenuation of the ultrasound signal [15], [16]. According to the criteria of the National Institutes of Health Clinical Research Network [17], [18], males with alcohol intake >140 g/week and females with alcohol intake >70 g/week were excluded. Patient with other causes of liver echogenicity, including viral, drug induced, or autoimmune hepatitis, were also excluded.

Diabetes mellitus was defined as fasting plasma glucose concentration >7.0 mmol/L [19], a previous diagnosis of DM, and/or HbA1c >6.5%.

### Statistical Analysis

Continuous variables are reported as mean (SD) and categorical variables as number (percentage). Between group differences were compared using *χ* ^2^ tests and t-tests, as appropriate.

Univariate and multivariate logistic regression models were used to assess the associations of risk factors with CAD, with results expressed as odds ratios (ORs) and 95% confidence intervals (CIs). Risk factors included in the multivariate logistic regression models were gallstone disease; patient age, gender, BMI, and waist circumference; serum TC, TG, LDL-C, and HDL-C, and fasting glucose concentrations; and history of hypertension, DM, NAFLD and MetS. Independent variables in two groups were ascertained by searching their medical records. Appropriate interaction terms were generated to test whether interactions were statistically significant.

To reduce the possible influence of selection bias, patients in the CAD-positive and CAD-negative groups were matched one-to-one by age, gender and MetS. Only these three covariates were chosen because 1) MetS already includes waist circumference; HDL-C, TG, and glucose concentrations; and blood pressure, important indicators of dyslipidemia, DM and hypertension; and 2) multivariable matching is normally not applied during matching. In addition, bias may have a significant effect on the results if a large number of subjects were lost after matching. Only patients with no missing values for all covariates were included.

All statistical analyses were performed using SAS version 9.1. All P values are two sided and P<0.05 was considered statistically significant.

## Results

**Figure 1:**
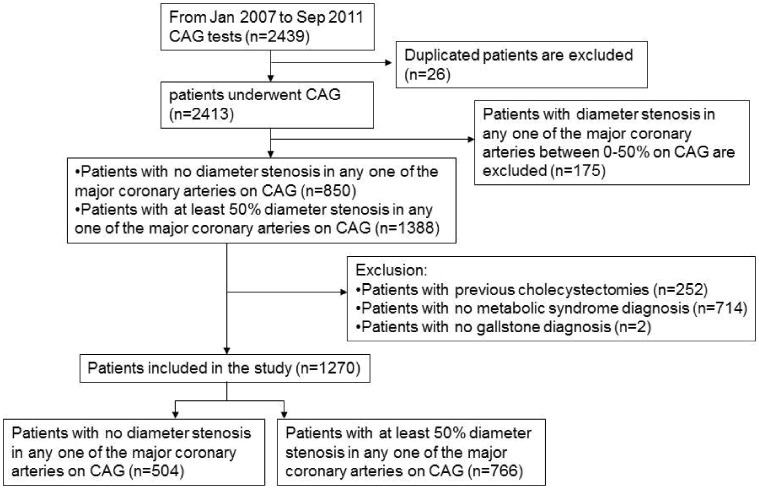
Study recruitment and participant flow. There were 2,413 patients who underwent coronary angiography for the first time included in the study. The final number of patients was 1,270 (766 in the CAD-positive and 504 in the CAD-negative group, respectively).

Figure 1 shows the study flow chart. We identified 2,413 patients who underwent coronary angiography for the first time from January 2007 to September 2011. After excluding patients with 0–50% diameter stenosis in any major coronary artery on coronary angiography (n = 175), those who had undergone previous cholecystectomy (n = 252), those with no clear MetS diagnosis (n = 714), and those with no gallstone diagnosis (n = 2), the study included 1,270 patients, 766 with and 504 without CAD.

The characteristics of patients in the CAD-positive and CAD-negative groups are shown in Table 1. The prevalence of gallstone disease was significantly greater in the CAD-positive than the CAD-negative group (149/766 [19.5%] vs 57/504 [11.3%], P<0.01). History of hypertension (78.5% vs 71.8%) and DM (41.3% vs 26.0%) were higher, and the prevalence of MetS was significantly higher (522/766 [68.2%] *vs* 238/504 [47.2%], P<0.01), in the CAD-positive than in the CAD-negative group. (Table 1).

**Table 1:** Demographic and clinical characteristics of the CAD-positive and CAD-negative groups.

	CAD-positive	CAD-negative	P value
No. of subjects	766	504	–
Gallstone disease (n, %)	149 (19.5)	57 (11.3)	<0.01
Age (years)*	67 (10.0)	62 (10.5)	<0.01
Male (n, %)	544 (71.0)	238 (47.2)	<0.01
BMI (kg/m^2^)*	24 (3.2)	24 (3.3)	0.73
Waist circumference (cm)*	86 (9.6)	84 (8.9)	<0.01
TC (mmol/L)*	4.2 (1.4)	3.8 (1.6)	<0.01
TG (mmol/L)*	2.2 (1.5)	2.6 (1.6)	<0.01
HDL-C (mmol/L)*	1.1 (0.3)	1.2 (0.5)	<0.01
LDL-C (mmol/L)*	2.7 (0.9)	2.6 (0.8)	0.63
Fasting glucose (mmol/L)*	6.0 (2.1)	5.3 (1.2)	<0.01
History of hypertension (n, %)	601 (78.5)	362 (71.8)	<0.01
DM (n, %)	316 (41.3)	131 (26.0)	<0.01
NAFLD (n, %)	363 (48.5)	237 (47.4)	0.70
MetS (n, %)	522 (68.2)	238 (47.2)	<0.01
MetS components (n, %)			
Waist circumference ≥90 cm for men or ≥80 cm for women	283 (37.0)	207 (41.1)	0.14
Fasting glucose >5.6 mmol/L	323 (42.2)	121 (24.0)	<0.01
TG≥1.7 mmol/L	426 (55.6)	304 (60.3)	0.09
HDL-C<1.0 mmol/L for men or <1.3 mmol/L for women	449 (58.6)	273 (54.2)	0.12

* data expressed as mean (SD).

Table 2 shows the unadjusted and adjusted ORs of risk factors associated with CAD. Univariate analysis showed that gallstone disease was significantly associated with CAD (unadjusted OR = 1.89, 95% CI: 1.36–2.63), as were older age, higher waist circumference, higher TC and fasting glucose concentrations, and the presence of hypertension, DM and MetS (Table 2). In multivariate analysis, after adjusting for all covariates, gallstone disease remained a significant risk factor for the development of CAD (adjusted OR = 1.59, 95% CI: 1.10–2.31). Older age, higher TC and fasting glucose concentrations, and diagnoses with DM and MetS were also associated with CAD (Table 2).

**Table 2:** Unadjusted and adjusted odds ratios of factors associated with CAD.

	Unadjusted OR	Adjusted OR
Gallstone disease	1.89 (1.36–2.63)**	1.59 (1.10–2.31)*
Age	1.05 (1.04–1.06)**	1.06 (1.05–1.07)**
Female gender	0.37 (0.29–0.46)**	0.23 (0.18–0.31)**
BMI	1.00 (0.96–1.03)	0.93 (0.87–1.00)
Waist circumference	1.03 (1.01–1.04)**	1.01 (0.99–1.04)
TC	1.24 (1.15–1.35)**	1.68 (1.44–1.95)**
TG	0.86 (0.80–0.93)**	0.86 (0.78–0.95)**
HDL-C	0.21 (0.14–0.32)**	0.18 (1.10–0.30)**
LDL-C	1.03 (0.91–1.17)	0.80 (0.63–1.00)
Fasting glucose	1.33 (1.22–1.46)**	1.23 (1.09–1.38)**
History of hypertension	1.43 (1.10–1.85)*	1.26 (0.94–1.69)
DM	1.99 (1.56–2.56)**	1.38 (1.05–1.83)*
NAFLD	1.05 (0.83–1.31)	0.97 (0.75–1.25)
MetS	2.39 (1.90–3.01)**	2.56 (1.94–3.36)**
MetS components		
Waist circumference ≥90 cm for men or ≥80 cm for women	0.84 (0.67–1.06)	0.99 (0.76–1.29)
Fasting glucose >5.6 mmol/L	2.31 (1.80–2.96)**	1.97 (1.47–2.65)**
TG≥1.7 mmol/L	0.82 (0.66–1.04)	0.87 (0.66–1.13)
HDL-C<1.0 mmol/L for men or <1.3 mmol/L for women	1.20 (0.96–1.50)	1.56 (1.19–2.04)**

* P<0.05;

** P<0.01.

Matching of patients in the CAD-positive and CAD-negative groups one-to-one by age, gender and MetS yielded 320 matched pairs. Table 3 shows their demographic and clinical characteristics. As in our main analysis, the prevalence of gallstone disease remained significantly higher in the matched CAD-positive than CAD-negative patients (66/320 [20.6%] vs 38/320 [11.9%], P<0.01). CAD-positive patients were also more likely to have higher TC, LDL-C and fasting glucose concentrations and to be diagnosed with DM (Table 3). Table 4 shows the unadjusted and adjusted ORs of risk factors in these matched pairs. Multivariate analysis continued to show that gallstone disease was significantly associated with CAD (adjusted OR: 1.69 95% CI 1.08–2.65), as well as with higher TC concentrations and a diagnosis of DM (Table 4).

**Table 3:** Demographic and clinical characteristics of the matched pairs in the CAD-positive and CAD-negative groups.

	CAD-positive	CAD-negative	P value
No. of subjects	320	320	–
Age (years)*	64 (9.8)	64 (9.8)	–
Male (n, %)	195 (60.9)	195 (60.9)	–
MetS (n, %)	186 (58.1)	186 (58.1)	–
Gallstone disease (n, %)	66 (20.6)	38 (11.9)	<0.01
BMI (kg/m^2^)*	24.3 (3.2)	24.7 (3.3)	0.16
Waist circumference (cm)*	85.3 (8.9)	85.7 (8.9)	0.62
TC (mmol/L)*	4.4 (1.3)	3.7 (1.5)	<0.01
TG (mmol/L)*	2.1 (1.7)	2.6 (1.6)	<0.01
HDL-C (mmol/L)*	1.1 (0.3)	1.1 (0.3)	0.06
LDL-C (mmol/L)*	2.8 (0.9)	2.6 (0.8)	<0.05
Fasting glucose (mmol/L)*	5.7 (1.7)	5.4 (1.4)	<0.05
History of hypertension (n, %)	239 (74.7)	247 (77.2)	0.46
DM (n, %)	122 (38.1)	98 (30.6)	<0.05
NAFLD (n, %)	162 (52.4)	156 (48.9)	0.38
MetS components (n, %)			
Waist circumference ≥90 cm for men or ≥80 cm for women	121 (37.8)	123 (38.4)	0.87
Fasting glucose >5.6 mmol/L	118 (36.9)	91 (28.4)	<0.05
TG≥1.7 mmol/L	160 (50.0)	203 (63.4)	<0.01
HDL-C<1.0 mmol/L for men or <1.3 mmol/L for women	179 (55.9)	187 (58.4)	0.52

* data expressed as mean (SD).

**Table 4:** Odds ratios of factors associated with CAD in matched pairs.

	Unadjusted OR	Adjusted OR
Gallstone disease	1.93 (1.25–2.98)**	1.69 (1.08–2.65)**
BMI	0.96 (0.91–1.02)	0.92 (0.85–1.00)
Waist circumference	1.00 (0.98–1.02)	1.00 (0.97–1.02)
TC concentration	1.38 (1.23–1.55)**	1.69 (1.90–2.06)**
TG concentration	0.81 (0.72–0.91)*	0.80 (0.71–0.90)*
HDL-C concentration	0.60 (0.35–1.01)	0.29 (0.14–0.57)*
LDL-C concentration	1.24 (1.03–1.48)*	0.85 (0.64–1.14)
Fasting glucose concentration	1.13 (1.01–1.26)*	1.10 (0.95–1.27)
History of hypertension	0.87 (0.61–1.25)	0.82 (0.56–1.20)
Diabetes mellitus	1.40 (1.01–1.94)*	1.42 (1.02–1.98)*
Non-alcoholic fatty liver disease	1.15 (0.84–1.58)	1.09 (0.80–1.50)
MetS components		
Waist circumference ≥90 cm for men or ≥80 cm for women	0.97 (0.71–1.34)	0.89 (0.64–1.25)
Fasting glucose >5.6 mmol/L	1.47 (1.05–2.05)*	1.42 (0.97–2.08)
TG≥1.7 mmol/L	0.58 (0.42–0.80)*	0.58 (0.41–0.81)*
HDL-C<1.0 mmol/L for men or <1.3 mmol/L for women	0.90 (0.66–1.24)	0.97 (0.69–1.37)

* P<0.05;

** P<0.01.

## Discussion

In this study, all the patients with and without CAD were diagnosed by coronary angiography. A significant association was observed between gallstone disease and CAD (adjusted OR = 1.59, 95% CI: 1.10–2.31), with this association confirmed by matched-pair analysis (adjusted OR = 1.69, 95% CI: 1.08–2.65).

Although previous studies assessed the association between cardiovascular disease and gallstone disease, their results were inconsistent [8]–[11]. In those studies, patients were not limited to CAD. More recently, two studies reported that cardiovascular disease, defined by ultrasonographic measurements of carotid artery intima-media thickness [13], and coronary heart disease, defined as myocardial ischemia using the excise treadmill test based on the Bruce protocol [20], were associated with gallstone disease. These diagnostic criteria, however, have several limitations. For example, their sensitivity and specificity were low for the detection of coronary heart disease by exercise electrocardiography, especially in women [21], [22]. Carotid artery intima-media thickness is an indirect marker of coronary heart disease. Thus, the percentage of patients with CAD in these two studies [13], [20], as well as in previous studies [9]–[11], [23], was unclear. Although these previous studies did not use the current gold standard (coronary angiography) for the diagnosis of CAD, they reported the association among gallstone disease and CAD. In our study, we used coronary angiography to differentiate CAD from other cardiovascular diseases. We were therefore able to exclude patients with other diseases that present with symptoms similar to CAD but with no obstruction in their coronary arteries. This resulted in a robust association between gallstone disease and CAD, as shown by the significantly higher prevalence of gallstone disease in CAD-positive than -negative patients (19.5% vs. 11.3%, p<0.01) and an adjusted OR of 1.59 for the association between these two conditions.

CAD and gallstone disease share many common factors, such as MetS [4], [24], DM [25], [26] and NAFLD [27], [28]. The liver is the key organ that regulates cholesterol metabolism and that controls plasma and biliary cholesterol concentrations [29], [30]. These pathways are impaired in patients with MetS, DM and fatty liver [31]–[34], leading to either dyslipidemia or supersaturation of biliary cholesterol. To reduce the possible influence of selection bias, patients in our CAD-positive and -negative groups were matched using three unbalanced variables, age, gender and MetS. Using this approach, we still found that gallstone disease *per se* was associated with CAD.

We also found that CAD and gallstone disease share several common risk factors such as higher total cholesterol and lower HDL cholesterol level. They are related with CAD even after matching for age, gender and MetS. The coincidence of the two diseases might be explained by the metabolic pathway of cholesterol in gut-liver axis [29], [30]. Interestingly, HDL cholesterol is reversely associated with both diseases. HDL plays a role in the reverse cholesterol transportation [35] and is uptake by its hepatic receptor – scavenger receptor B type I (SRBI). Increased expression of hepatic SRBI is found in Chinese patients with gallstone disease [36], which may provide the origin of excessive cholesterol for biliary secretion [37], [38] and leadw to gallstone formation. Moreover, higher fasting glucose level is the other factor associated with CAD, as well as gallstone disease [39], [40]. Higher fasting glucose or presence of DM may affect the metabolism of cholesterol and lipoprotein and cause defect in the gallbladder contractility [41], [42].

Between January 2007 and September 2011, 2413 patients underwent CAG at our center. However, after applying our exclusion criteria, only 1270 patients were included in the final analysis. Thus, nearly 50% of the patients who underwent CAG were excluded from the study, primarily because the presence or absence of MetS was not determined in around 30% of patients. We found that the demographic and clinical characteristics of the included and excluded subjects were similar, indicating that the included participants were representative of the total population and suggesting that bias was not introduced by our exclusion criteria.

We cannot, however, ignore the influence of genetic predisposition to both diseases. CAD and gallstone disease are complex diseases affected by several genetic factors, including polymorphisms in the genes encoding apolipoprotein E [43], [44] and the ATP binding cassettes G5 and G8 [45], [46]. Unfortunately, we were not able to obtain the family history of both diseases from our patients or genotypic information about certain genes. Thus, we could not evaluate the possible contributions of genetic factors to CAD and gallstone disease in our patient cohort.

The major limitation of this study was its cross-sectional design. Although we showed an association between gallstone disease and CAD, we could not determine causality. Longitudinal and mechanistic studies are needed to assess this association. In addition, although patient matching reduced selection bias to some extent, a Neyman bias may have affected our results. Furthermore, as yet undetermined confounders may have influenced the relationship between gallstone disease and CAD.

In conclusion, we observed a significant association between gallstone disease and CAD. An 18-year follow-up study found that gallstone disease was significantly associated with increased mortality from cardiovascular disease [47]. Together, these associations indicate that patients with gallstones should be monitored for the development of CAD.
